# Latin American and Caribbean contributions to oral pathology/medicine journals: Cross-sectional study

**DOI:** 10.4317/medoral.27934

**Published:** 2026-04-19

**Authors:** Samuel Trezena, Luiz Miguel Ferreira, João Pedro Santos Nascimento, Alice Maria Campos Fernandes Santos, Fabrício Emanuel Soares-de Oliveira, Alan Roger Santos-Silva, Daniella Reis Barbosa Martelli, Hercílio Martelli-Júnior

**Affiliations:** 1 Department of Dentistry, Center for Biological and Health Sciences, State University of Montes Claros, UNIMONTES, Montes Claros, Minas Gerais, Brazil; 2 Postgraduate Program in Health Sciences, Center for Biological and Health Sciences, State University of Montes Claros, UNIMONTES, Montes Claros, Minas Gerais, Brazil; 3 Department of Oral Diagnosis, School of Dentistry, University of Campinas, FOP-UNICAMP, Piracicaba, São Paulo, Brazil; 4 Department of Mental Health and Public Health, Psychology Course, Center for Biological and Health Sciences, State University of Montes Claros, UNIMONTES, Montes Claros, Minas Gerais, Brazil; 5 Postgraduate Program in Primary Health Care, Center for Biological and Health Sciences, State University of Montes Claros, UNIMONTES, Montes Claros, Minas Gerais, Brazil

## Abstract

**Background:**

To analyze the representation of Latin American and Caribbean (LAC) countries in international journals of Oral Pathology and Oral Medicine (OP/OM), focusing on authorship and editorial board participation.

**Material and Methods:**

Cross-sectional scientometric study. Editorial board data were extracted from six OP/OM journals. Publications indexed in Web of Science (1978-2025) were analyzed to identify contributions from LAC-affiliated researchers.

**Results:**

Only 13.2% of 410 editorial board members were affiliated with LAC institutions, mostly from Brazil (8.3%). Among 27,188 articles, 3,734 (13.7%) had LAC-affiliated first or senior authors, generating 53,588 citations (11.3%). A strong correlation was found between editorial participation and publication volume (=0.92; p=0.008). Oral Diseases had the highest share of LAC publications (22.1%), while Medicina Oral, Patología Oral y Cirugía Bucal had the highest citation percentage from LAC (27.2%). Scientific output was concentrated in Brazil (75.7%), while 11 LAC countries had no publications.

**Conclusions:**

Contributions to OP/OM journals from LAC have increased, particularly in Brazil and Mexico. However, editorial under-representation and regional disparities persist, especially among smaller and Caribbean countries. Expanding editorial diversity and strengthening regional collaborations are essential strategies for amplifying LAC's participation in the global PO/OM research.

## Introduction

In 2022, researchers from 23 Latin American countries contributed to 2,321 dental science publications, with Brazil alone accounting for 73.15% of this output (1). This surge reflects the region's increasing engagement in global scientific discourse. However, despite this upward trend, disparities persist in the visibility and impact of research from LAC countries (2).

Editorial boards play a pivotal role in shaping the scientific narrative, influencing publication decisions, and setting research agendas (3 , 4). Yet, a significant underrepresentation of LAC scholars on these boards has been documented (5 , 6). For instance, only 5% of editorial members in global health journals hail from Latin America and the Caribbean, highlighting a broader pattern of geographic imbalance (6). Such disparities can lead to biases in topic selection and hinder the inclusion of diverse perspectives, ultimately affecting the global relevance and equity of scientific research (7).

In Oral Pathology (OP) and Oral Medicine (OM), this underrepresentation is particularly concerning (8 , 9). While Brazil has made notable strides, contributing significantly to international research, and occupying editorial positions (10 - 13), other LAC countries remain largely absent from high-impact journals. Barriers such as limited access to research funding, language constraints, and institutional challenges further exacerbate this issue. Despite the existence of studies focusing on Brazilian contributions (14), there is a paucity of comprehensive analyses encompassing the broader LAC region and examining both authorship and editorial representation.

Addressing this gap is crucial for fostering a more inclusive and representative scientific community in OP/OM (15). Understanding the extent of LAC participation in high-impact journals can inform strategies to enhance visibility, promote equity, and support the integration of diverse voices in global research. Therefore, this study aims to analyze the representation of LAC in the international scientific literature in OP and OM.

## Material and Methods

Study Design

This cross-sectional bibliometric/scientometrics analysis was structured in two complementary phases: Analysis of LAC representation on editorial boards in OP/OM journals; and evaluation of scientific publications from these regions in the same journals. All data used in this study were obtained from publicly available sources.

Six specialized journals in OP/OM were selected based on the following inclusion criteria: A primary scope focused on Oral Pathology and Oral Medicine; consistent inclusion in prior thematic bibliometric analyses (10); and a high impact factor ranking within the top 100 of the 'Dentistry, Oral Surgery &amp; Medicine' category of the Journal Citation Reports (JCR): Head and Neck Pathology, Journal of Oral Pathology &amp; Medicine, Medicina Oral, Patología Oral y Cirugía Bucal, Oral Diseases, Oral Oncology, and Oral Surgery, Oral Medicine, Oral Pathology, Oral Radiology. These journals represent the most influential and representative peer-reviewed outlets for the specialty. The first phase focused on identifying editors affiliated with institutions in LAC, as defined by the United Nations geoscheme. The second phase analyzed publications authored by researchers from these regions that were indexed in the Web of Science (WoS) database. Data collection was carried out between January and April 2025.

Data Collection Procedures

Three researchers (JPSN, LMF, and ST) independently performed manual extraction of the names and roles of all editorial board members from each journal's official website. The following variables were collected for each editor: Full name, gender, editorial role, and country of institutional affiliation. Institutional data were verified using publicly available academic profiles (Google Scholar®, ResearchGate®, ORCID®) and official websites of universities and research institutions. Discrepancies were resolved through consensus.

In the second phase, the WoS database was used to extract all indexed scientific articles from the six selected journals. No time-related exclusion criteria were applied; therefore, all publications available in the database since its inception were included, covering the historical period from 1978 to 2025. Records were filtered using the "Country" field to identify articles with at least one author affiliated primarily with an institution in LAC. In multicentric studies and international collaborations, the publication was attributed to the LAC region only when the first author or the senior author (defined as the last author in the sequence) held a primary LAC affiliation. In cases of authors with dual affiliations, the primary institution listed in the manuscript was considered. All classification processes and potential discrepancies regarding institutional affiliation were resolved through consensus among the three researchers. Variables collected included article title, year of publication, corresponding author's country, and number of citations. To minimize sampling bias, all indexed articles were included regardless of language or document type. Classification bias was mitigated through triangulation by the three researchers during the collection of affiliation and gender data. Temporal trends were interpreted cautiously to avoid misrepresentation of fluctuations in scientific productivity.

Statistical Analysis

All data were consolidated and imported into IBM SPSS® Statistics version 27.0 (IBM Corp., Armonk, NY, USA) for descriptive and inferential analysis. Categorical variables were expressed as absolute and relative frequencies. For continuous variables, mean, standard deviation, median, and interquartile range were calculated according to data distribution. The Kolmogorov-Smirnov test (p&lt;0.05) confirmed non-normal distribution, justifying the use of non-parametric tests. Associations between categorical variables were assessed by Pearson's Chi-square test. Correlation between the number of publishers from LAC countries and the volume of publications was analyzed by Spearman's correlation (). The strength of the correlation was &lt;0.3 (weak), 0.3 &lt;0.7 (moderate), 0.7 (strong). Comparisons of the number of citations between journals and countries were performed using the Kruskal-Wallis test, complemented by Dunn's post-hoc analyses with Bonferroni adjustment when applicable.

## Results

Representation on editorial boards

Analysis of editorial board composition revealed 410 members. A male majority was observed (68%), with the highest regional representation from North America (34.4%, excluding Mexico), followed by Europe (32%). LAC countries constituted 13.2% of editorial board members. The LAC nations represented and their respective proportions were: Brazil (8.3%), México (2.2%), Chile (0.7%), Uruguay (0.7%), Peru (0.5%), Costa Rica (0.2%), Guatemala (0.2%), and Venezuela (0.2%). Notably, only 24.2% of LAC countries had representation on editorial boards. Regarding editorial roles, a single member from Brazil served as Editor-in-Chief, while 17 members (4.1%) held Associate Editor positions. Journal-specific representation varied substantially, with Head and Neck Pathology exhibiting the lowest LAC representation (a single member from Brazil, constituting 0.2% of editorial board members), while Medicina Oral, Patología Oral y Cirugía Bucal demonstrated the highest representation (28 researchers, accounting for 6.8% of total members). The association between LAC representation and member/journal characteristics is presented in Table 1.

Table 1Representation in scientific production

The WoS search retrieved 27,188 publications with 474,485 citations. Of these, 3,734 publications (13.73%) were affiliated with LAC countries, receiving 53,588 citations (11.29%). The mean citations per publication was 17.45 (±41.29) overall, while LAC-contributed publications showed a lower mean of 14.35 (±23.76). Spearman's correlation analysis revealed a strong positive association between the number of editorial board members and the number of publications originating from these countries (=0.92, p=0.008).

Oral Diseases is the journal with the highest number of publications by LAC authors, totaling 825 scientific articles (22.1%), followed by 691 (18.5%) in Medicina Oral, Patología Oral y Cirugía Bucal, 669 (17.9%) in Oral Surgery, Oral Medicine, Oral Pathology, Oral Radiology, 662 (17.7%) in the Journal of Oral Pathology &amp; Medicine, 637 (17.1%) in Oral Oncology, and 250 articles (6.7%) in Head and Neck Pathology. While Medicina Oral, Patología Oral y Cirugía Bucal demonstrated the highest proportion of citations attributed to LAC publications (27.23%), Oral Oncology recorded both the highest total citation count (170,424) and a superior mean citation rate per article (18.34±31.85). In contrast, Head and Neck Pathology showed the lowest LAC contribution proportion (8.45%) and the lowest mean citation rate (9.16±13.46), while Journal of Oral Pathology &amp; Medicine achieved the second-highest mean citations (17.90±23.15), closely following Oral Oncology. These inter-journal differences were statistically significant (p&lt;0.001) (Table 2).

Table 2LAC publications were published between January 1978 and April 2025, with an exponential annual increase since the last decade. The years 2022 and 2024 have the highest number of publications (280 and 355, respectively).

**Figure 1:**
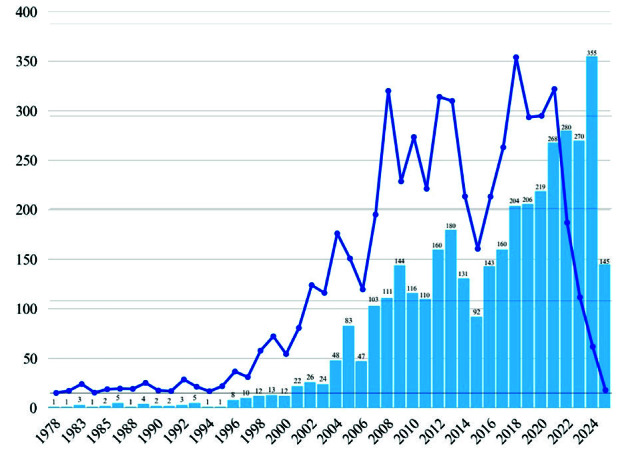
Historical series of publications (bars) and citations (line) from Latin American and Caribbean (LAC) countries in Oral Pathology and Oral Medicine journals.

**Figure 2:**
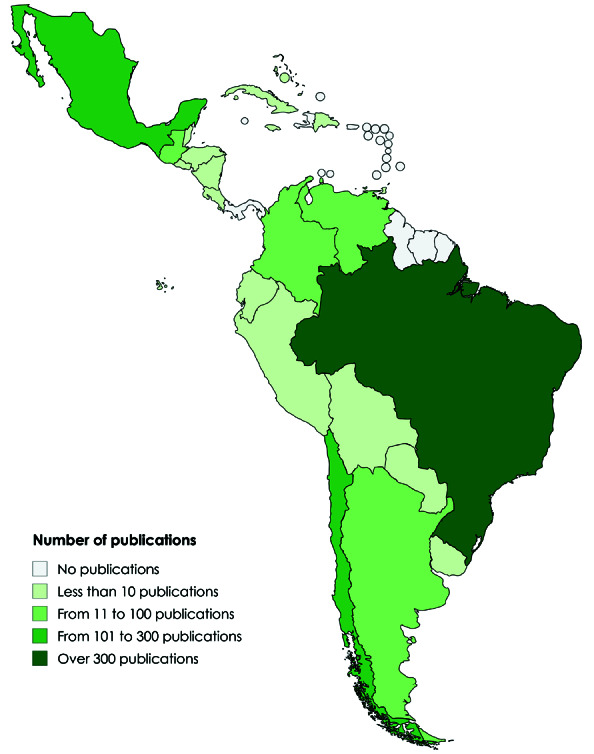
Distribution of scientific publications by country in Latin America and the Caribbean (LAC).

Regarding citations, an increasing trend was observed from 2015 onwards, with the peak being reached in publications in 2018 (Figure 1). Figure 2 presents the map showing the distribution of publications by country.1,2

Among the 33 LAC countries, no publications were identified for 11 nations (33.3%): Panama, Antigua and Barbuda, Barbados, Dominica, Grenada, Haiti, Saint Lucia, Saint Kitts and Nevis, Saint Vincent and the Grenadines, Guyana, and Suriname. Brazil ranks first with 2,825 articles (75.7%), followed by Mexico with 281 (7.5%), Chile with 146 (3.9%), and Argentina with 116 (3.1%). Cuba demonstrated exceptional citation impact with the highest mean citations per article (36.80±60.50), despite contributing only 10 publications (0.3%). Conversely, El Salvador recorded the lowest mean citations (2.33±1.52) among countries with 3 publications. Guatemala showed strong performance with 72 publications achieving high mean citations (20.13±23.99). Significant disparities in citation metrics were confirmed across countries (Table 3).

Table 3

## Discussion

This is the first study that assessed the detailed participation of LAC countries in OP/OM journals. The analysis presented here revealed complex patterns of scientific production and editorial representation in LAC. The results demonstrate significant advances in recent decades, but also deep structural disparities that may be reflecting socioeconomic asymmetries, fragmented science policies, and systemic barriers to international integration (16).

Our analysis reveals that Latin American researchers constitute merely 13.2% of editorial board members, with 75.8% of LAC countries wholly unrepresented in these decision-making bodies. Previous studies have shown that representatives from low- and middle-income countries have difficulty in serving on editorial boards (5 , 17 , 18). This structural underrepresentation undermines the theoretical benefits of diversity in practice, perpetuating geopolitical biases in knowledge curation, hindering the reduction of linguistic and cultural barriers for local researchers, and limiting the incorporation of urgent regional issues into the scientific mainstream (18). Thus, the observed quantitative and geographic gap not only reflects entrenched academic hierarchies, but actively and concretely obstructs the construction of a globally relevant, diverse, and contextualized science (18 , 19).

The underrepresentation of Latin American on editorial boards may be intrinsically linked to the relatively recent consolidation of OM as a formal specialty in these countries, a factor that directly impacts the number of researchers meeting international standards (8 , 20 , 21). This scenario contributes to a vicious cycle in which limited regional scientific output leads to reduced academic visibility and, consequently, fewer invitations to participate in editorial roles.

Head and Neck Pathology's minimal LAC editorial presence and low LAC publications may be interrelated variables. A previous study evaluating publication patterns in oral health journals confirmed that editorial board members exhibit higher publication rates in their affiliated journals (22), suggesting that underrepresentation perpetuates barriers to regional research submission. On the other hand, Medicina Oral, Patología Oral y Cirugía Bucal demonstrates the highest LAC editorial representation among analyzed journals. This representation may be linked to the "nationality" of the journal. "Med Oral" is a Spanish journal (jcr.clarivate.com) and, due to its language like that spoken in Latin countries, it may favor greater exchange between nations and, consequently, invitations to participate in the editorial board. Global studies confirming that editorial board diversity predicts geographic inclusion (4 , 5). Oral Oncology was the journal with the highest number of citations. This finding may be directly linked to the significant increase in recent years in research related to Oral Cancer (22).

The significant difference in scientific production among LAC countries is generally associated with structural factors, such as investment in research, contingent of qualified postgraduate programs (PPG) and socioeconomic indicators. Furthermore, social indicators of education are related to the academic robustness of a community. Therefore, countries with fewer strong programs, less financial support and low institutional capacity tend to have lower production in science. Evidenced in previous research that shows a Latin America with incipient PPG focused on OP/OM (9 , 23). The exponential growth of Brazilian scientific output since the 1990s is largely attributable to national postgraduate policies. Initiatives like the Third National Postgraduate Plan (PNPG) expanded doctoral programs and research infrastructure, while subsequent plans introduced key measures such as the CAPES Journal Portal and internationalization efforts (https://www.gov.br/capes/pt-br/centrais-de-conteudo/documentos/19122023_pnpg_2024_2028.pdf). More recently, this progress has been reinforced by strategic federal programs. The Institutional Internationalization Program (PrInt), the Postgraduate Partnership Program (PEC-PG), and the massive Science Without Borders program, which invested over two billion USD in international mobility for students and researchers, have been pivotal. Coordinated by agencies like CAPES and CNPq, these efforts have consolidated a robust scientific system, significantly strengthening Brazil's global research presence and international collaboration (13 , 24). In Mexico, the Program for Professional Teacher Development PRODEP (https://dgesui.ses.sep.gob.mx/programas/programa-para-el-desarrollo-profesional-docente-para-el-tipo-superior-s247-prodep) aims to expand and qualify teacher training, has encouraged international collaborations, and has demonstrated a significant impact on scientific productivity by promoting the professionalization of full-time teachers and the formation of organized research groups.

Furthermore, national programs to support academic "mobility" have increased the exchange of students and researchers, which may have influenced larger networks of collaborations and partnerships in research (25 , 26). A bibliometric study on Brazil's international scientific collaborations showed that publications with partnerships significantly increase the number of citations in high-impact journals. In addition, well-defined collaboration networks were observed with European countries and another with Latin American countries (13).

The strong dependence of Latin American countries on research agendas from the global North reinforces critical debates about regional scientific hegemony and autonomy. A study on CONICET in Argentina highlights this phenomenon (27). Although there is some space for local themes in research, most of the production follows the dominant international paradigms, which indicates a form of knowledge colonialism and academic extractivism, where hegemonic centers capture the fruits of peripheral science without guaranteeing epistemological autonomy (27).

While Brazil's output signifies scientific maturation, it also exposes regional fragmentation. Smaller nations lack equivalent PPG density, stable funding, and institutional support for manuscript submission. This creates a "Brazil-centric" LAC profile, inadvertently marginalizing neighboring research ecosystems. Eleven Caribbean nations contributed zero publications, a consequence of underfunded health research systems, limited research infrastructure, insufficient human resources, and the absence of institutional incentives to promote scientific outputs (27). Scientific production in Caribbean countries may be also associated with the lower number of specialists in the area. A previous study found that these countries have a small number of OM specialists, and that almost half of the countries do not have specific regulations regarding the understanding of clinical competencies (9). Nonetheless, disparities persist, and building a sustainable scientific ecosystem in the Caribbean will require coordinated investment in capacity building, visibility strategies, and cross-border collaboration.

As limitations of this study, we can mention the possible exclusion of LAC researchers with dual affiliation or external subsidy. Furthermore, only journals with a scope in OP/OM were selected, excluding journals in Dentistry that also publish manuscripts on the subject. A critical factor in interpreting our findings is the 'citation-time bias.' Since total citation counts were not normalized by publication year, older manuscripts naturally exhibit higher absolute citation numbers due to their extended period of exposure and accumulation within the scientific community. This is particularly relevant considering the exponential growth of LAC publications in OP/OM observed over the last decade. While recent contributions from the region show high contemporary relevance, their total impact metrics are still maturing. Consequently, the scientific influence of LAC researchers may be even more significant than absolute numbers suggest, as a substantial portion of the region's production consists of recent, high-quality studies that have not yet reached their citation peak.

The simultaneous use of two checklists underscores the need for new guidelines tailored to scientometric studies, as innovations in the field still lack appropriate reporting standards. However, this study brings broad perspectives on the need to draw attention to the inclusion of developing countries in the high echelons of scientific research.

## Conclusions

Although the overall picture points to notable advances in the visibility of OP/OM research in LAC, structural gaps remain that limit the full integration of smaller and Caribbean countries. Democratizing access to publishing networks and promoting multilateral partnerships, associated with national policies for academic development and training, are essential measures to ensure that regional diversity is reflected equally in global scientific discourse.

## Figures and Tables

**Table 1:** Distribution of Latin American and Caribbean countries according to the characteristics of editorial board members and journals.

	Latin America and the Caribbean		
	Yes	No	
Variables	N (%)		p-value*
Gender			0.241
Male	33 (11.8)	246 (88.2)	
Female	21 (16.0)	110 (84.0)	
Journal			<0.001*
Head and Neck Pathology	1 (2.1)	46 (97.9)	
Journal of Oral Pathology & Medicine	7 (12.1)	49 (87.5)	
Medicina Oral, PatologÃ­a Oral y CirugÃ­a Bucal	28 (31.5)	61 (68.5)	
Oral Diseases	9 (14.8)	52 (85.2)	
Oral Oncology	2 (2.0)	97 (98.0)	
Oral Surgery, Oral Medicine, Oral Pathology, Oral Radiology	7 (12.1)	51 (87.9)	
Editorial function			0.263
Editor-in-Chief	1 (12,5)	7 (87.5)	
Deputy Editor	-	3 (100.0)	
Advisory Editor	-	20 (100.0)	
Associate Editor	17 (18.9)	73 (81.1)	
Section Editor	-	2 (100.0)	
Editorial Board	36 (12.5)	251 (87.5)	

*Significant statistical association with Pearson's chi-square test (p<0.05).

**Table 2:** Citation’s metrics in Oral Pathology and Oral Medicine journals.

	Citations			
Journal	Total	From LAC N (%)	Mean±SD	p-value
Head and Neck Pathology	27,069	2,289 (8.45)	9.16±13.46	<0.001*
Journal of Oral Pathology & Medicine	119,98	11,850 (9.87)	17.90±23.15	
Medicina Oral, PatologÃ­a Oral y CirugÃ­a Bucal	35,202	9,589 (27.23)	13.88±19.53	
Oral Diseases	82,508	11,326 (13.72)	13.73±27.70	
Oral Oncology	170,42	11,680 (6.85)	18.34±31.85	
Oral Surgery, Oral Medicine, Oral Pathology, Oral Radiology	39,306	6,854 (17.43)	10.25±13.98	

LAC: Latin American and Caribbean. *Significant statistical association with Kruskal-Wallis test (p<0.05).

**Table 3:** Citations metrics by Latin American and Caribbean countries contributions in Oral Pathology and Oral Medicine journals.

	Publications	Citations		
Country	N (%)	Total	Mean±SD	p-value
Brazil	2,825 (75.7)	39,23	13.89±23.49	0.002*
Argentina	116 (3.1)	1,947	16.78±20.27	
Chile	146 (3.9)	2,355	16.13±26.75	
Colombia	75 (2.0)	1,227	16.36±26.33	
Ecuador	9 (0.2)	38	4.22±3.96	
Guatemala	72 (1.9)	1,449	20.13±23.99	
Mexico	281 (7.5)	4,451	15.84±23.94	
Paraguay	9 (0.2)	48	5.33±5.45	
Peru	47 (1.3)	759	16.15±22.21	
Uruguay	64 (1.7)	770	12.03±29.25	
Venezuela	44 (1.2)	561	12.75±17.92	
Costa Rica	9 (0.2)	118	13.11±18.36	
Cuba	10 (0.3)	368	36.80±60.50	
Trinidad and Tobago	9 (0.2)	99	11.00±18.85	
Belize	5 (0.1)	99	19.80±21.02	
Bolivia	3 (0.1)	12	4.00±3.60	
El Salvador	3 (0.1)	7	2.33±1.52	
Jamaica	3 (0.1)	35	11.67±16.82	
Bahamas	1 (0.0)	1	-	
Honduras	1 (0.0)	4	-	
Nicaragua	1 (0.0)	4	-	
Dominican Republic	1 (0.0)	4	-	

*Significant statistical association with Kruskal-Wallis test (p<0.05).

## Data Availability

Declared none.
